# 

**Published:** 1835-10-01

**Authors:** 


### BIBLIOGRAPHICAL RECORD;

OR,

*Works received for Review since last Quarter.*

1. Dublin Journal of the Medical and Chemical Sciences, &c. July, 1835.

2. Illustrations in British Zoology. By George Johnston, M.D.—(From the Magazine of Natural History, vol. viii.)

3. A further Inquiry concerning Constitutional Irritation, and the Pathology of the Nervous System. By Benjamin Travers, F.R.S. Senior Surgeon to St. Thomas's Hospital. One Vol. 8vo. pp. 444. Longman and Co. 1835.

4. An Essay on the Nature of Diseases. By A. Green, LL.B. Octavo, pp. 51. Simpkin and Marshall, July, 1835. 2s.

5. Lectures on the Diseases of the Lungs and Heart. By Thomas Davies, M.D. Physician to the Infirmary for Asthma, Consumption, &c. Octavo, pp. 512. Longman and Co. July, 1835.

6. A new Practical Formulary of Hospitals of England, Scotland, Ireland, France, Germany, Italy, Spain, Portugal, &c. &c.; Translated from the French of Milne Edwards and P. Vavasseur, and considerably augmented, by Michael Ryan, M.D. Duodecimo, pp. 544. Henderson, London, July, 1835.

☞ *This is one of the most valuable little vade-mecums that we have ever seen. It is indispensable for the practitioner of every grade and denomination.*

7. The Clinique Médicale; or Reports of Medical Cases. By G. Andral. Condensed and Translated by Dr. Spillan. Part II. Diseases of the Chest. Pp. 240. Renshaw, July, 1835.

8. A Treatise on the more obscure Affections of the Brain, on which the Nature and successful Treatment of many Chronic Diseases depend, &c. By A. P. W. Philip, M.D. &c. Octavo, pp. 140. Renshaw, July, 1835.

9. The Second Biennial Report of the London Ophthalmic Infirmary, &c. Octavo, pp. 37. 1835.

10. The American Cyclopædia of Practical Medicine and Surgery—a Digest of Medical Literature. Edited by Isaac Hays, M.D. Part VII. "Anus" to "Apoplexy." Philadelphia, April, 1835.

11. A Lecture introductory to the Science of Comparative Anatomy. By H. W. Rush, M.R.C.S. Pp. 16. 1835.

12. An Address to the Liverpool Medical Society, on being elected one of its Fellows. By Thomas Jeffreys, M.D.

☞ *Contains several hints, well adapted for a medical society.*

13. A Practical Treatise on Diseases of the Teeth, in which the Origin and Nature of Decay are explained, and the Means of Prevention pointed out. By William Robertson. Birmingham, July, 1835.

☞ *In our next.*

14. A Treatise on Pulmonary Consumption, comprehending an Inquiry into the Causes, Nature, Prevention, and Treatment of Tuberculous and Scrofulous Diseases in general. By James Clark, M.D. F.R.S. Physician in Ordinary to their Royal Highnesses the Duchess of Kent and the Princess Victoria. Octavo, pp. 399. London, July, 1835.

☞ *This is an enlargement of the able article in the Cyclopædia of Practical Medicine.*

15. Stammering considered, with respect to its Cure by the Application of those Laws which regulate Utterance. By Richard Cull. Octavo, sewed, pp. 52. Renshaw, July, 1835.

16. Report (Parliamentary) from the Select Committee on Medical Education, with the Minutes of Evidence and Appendix. Part I. Royal College of Physicians —Part II. Royal College of Surgeons. Presented by Henry Warburton, Esq.

17. Oratio ex Harveii Instituto habita Œdibus Collegii, &c. die Junii 25, 1835. Ab Henrico Halford, M.D. &c.

18. Practical Observations on the Nature and Treatment of Nervous Diseases; with Remarks on the Efficacy of Strychnine in the more obstinate Cases. By Geo. Russell Mart, M.R.C.S. late of His Majesty's Hospital-Ship Racoon. Octavo, pp. 185. Churchill, July, 1835.

---

19. The Principles and Practice of Obstetric Medicine, &c. By Dr. D. Davis. Part 42, with Plates. August, 1835. Price One Shilling.

☞ *This Part contains* 48 *quarto pages of letter-press and four Plates—all for a shilling!*

---

20. A Series of Anatomical Plates in Lithography, &c. By Dr. Jones Quain. Fasciculi 26 and 27. Division 2, Arteries 2 and 3.

☞ *The fourth Plate, representing the arteries about the neck and axilla, is remarkably well executed.*

---

21. An Introduction to the Study of Practical Medicine; being an Outline of the leading Facts and Principles of the Science, as taught in a Course of Lectures delivered in the Mareschal College, Aberdeen. By John M'Robin, M.D. &c. Octavo, pp. 226. Part the First.

☞ *This Work is designed as a text-book for students, and appears to be very judiciously compiled, with the view of assisting the tyro in the acquisition of elementary knowledge.*

---

22. The Dublin Journal of Medical and Chemical Science, &c. Sept. 1835.

☞ *In exchange.*

---

23. The Medical Student's Practical and Theoretical Guide to the Translation and Composition of Latin Prescriptions; with an explanatory Latin Grammar, &c. Small 8vo, pp. 170. By J. W. Underwood. Souter, August, 1835, price 5s. 6d.

---

24. A Critical Analysis of the Evidence adduced at the Inquest, occasioned by the late Explosion at Wallsend Colliery, &c. By George Fife, M.D. pp. 45. Newcastle-on-Tyne, Sept. 1835.

---

25. Formulary for the Preparation and Employment of several new Remedies, such as Morphine, Codeine, &c. Translated from the eighth Edition of the Formulaire of M. Magendie, with an Appendix, by Chas. Wilson Gregory, M.D. Royal 8vo, pp. 225. Cox, St. Thomas-st. Borough. August, 1835.

☞ *This translation is somewhat more copious than Dr. Gully's, from which an extract will be found in our pages (Kreosote); but, on comparing several articles, we find no material difference. The price differs by Two Shillings.*

---

26. A Map of the Anatomy of the Eye, with the latest Discoveries. By J. H. Curtis, Esq. Oculist and Aurist. Price Five Shillings.

☞ *The numerous figures in this plate are extremely well calculated to facilitate the study of that complicated, but beautiful apparatus—the* HUMAN EYE.

---

27. A Treatise on Hydrocephalus, or Water in the Brain; with the most successful Modes of Treatment. By William Griffith, M.R.C.S. Small 8vo, pp. 86. August, 1835.

---

28. A Manual of Select Medical Bibliography, in which the Books are arranged chronologically, according to the Subjects, and the Derivations of the Terms, and the Nosological and Vernacular synonyms of the Diseases are given, with an Appendix, &c. &c. By John Forbes, M.D. Royal 8vo, pp. 403. Double Columns. August, 1835.

☞ *This is a re-publication, with additions, of the Bibliographical Supplement to the Cyclopædia of Practical Medicine, and does infinite credit to the laborious research of the author.*

---

29. A Dictionary of Terms used in Medicine and the Collateral Sciences. By Richard D. Hoblyn, M.A. Oxon. 8vo. pp. 329. Double Cols. Price 9s. Sherwood and Co.

☞ *Concise and ingenious.*

---

N. B.—The Bibliographical Record of this Number closed on the 18th of September.

---

Errata.

In page 284 of our last Number, *for* Dr. Fleming *read* Dr. Heming—and *for* F. Cumin, *read* Dr. Cumin.

### INDEX.

A.

Abdomen, Dr. Macfarlane's report on tumors of the ........ 254

Abdomen, cystic sarcoma of the .... 256

Abdomen, encysted tumor of........ 258

Abdomen, organized sarcoma of .... 259

Abdomen, fibro-cartilaginous tumor of 259

Abdomen, injuries of ............. 526

Abscess in lungs, supposed to be cured, 213

Academy, French, its contempt for homœopathy ........ 239

Acalephæ ........ 284

Acrodyniæ, account of the. ......... 424

Alison, Dr. on the arteries of inflamed parts ........ 497

Alison, Dr. address to the Irish profession ........ 511

Alvine concretions, tumors from .... 263

Amaurosis, Mr. Middlemore on...... 254

Amputation, partial, of the foot .... 499

Andral, M. his Clinique Medicale.... 89

Andral, M. on gastro-enteritis ...... 216

Andral, M. his Clinique Medicale.... 345

Aneurism, thoracic, use of the stethoscope in........ 477

Aneurism by anastomosis, Mr. Adams on ........ 511

Animals, their history, &c. as shewing the wisdom of God........ 400

Animals, creation of ........ 401

Antimony in acute rheumatism...... 235

Antimony for tetanus ........ 474

Anus, sloughing of the ........ 527

Aortic aneurism, interesting case of.. 190

Apothecaries' curriculum, last ...... 189

Apothecaries' Company, curriculum of 289

Arteries of inflamed parts ........ 497

Arthritis, report on ........ 195

Asphyxia, Dr. Alison on ........ 498

Associations for protecting medical practitioners ........ 285

Atlas of cutaneous diseases ........ 578

Auscultation of the heart ........ 77

Auscultic theories of the heart's action ........ 78

Auscultic phenomena of heart's action, 80

B.

Babington's epidemics of middle ages.. 49

Badham, Dr. on the effects of inordinate depletion........ 475

Banff Medical society........ 579

Bartholomew's Hospital, circular of the officers of ........ 162

Bellis, Mr. his case of aortic aneurism, 190

Biliary ducts, occlusion of the ...... 452

Birmingham Infirmary, annual report of........ 241

Birmingham Eye Infirmary, annual report of ........ 251

Blackley, Mr. on erythema nodosum.. 466

Bloodletting in pneumonia, M. Bouillaud on ........ 200

Bones in the heart of the ox ........ 495

Bostock's history of medicine ...... 53

Bouillaud, M. abstract of his clinical course ........ 183

Brain, cases of disease of the........ 90

Brain, M. Andral on hyperæmia of the, 96

Brain, Dr. Philip on diseases of the .. 515

Bridgewater Treatise, Dr. Kirby's.... 400

Bristol Infirmary (clinical) ........ 531

British Association (fifth meeting) .. 489

British Association, conclusion of, in Dublin ........ 511

Bronchial glands, diseases of the .... 76

Bruit de soufflet, Dr. Corrigan on.... 505

C.

Cæsarean operation, successful...... 505

Callus, absorption of, in fracture .... 523

Carbutt, Dr. his clinical report on dropsy ........ 272

Carbutt, Dr. on diabetes ........ 542

Carditis, Dr. Stroud's cases of ...... 441

Carmichael, Mr. on the cause of sleep, 470

Carswell, Dr. his pathological anatomy, 122

Catalepsy, curious case of.......... 145

Cataract, Mr. Middlemore on ....... 253

Cataract, Mr. Watson on .......... 568

Cephalopods, Mr. Kirby on the... .. 409

Cerebellum completely absent ...... 141

Cerebral hæmorrhage, M. Andral on.. 345

Cerebral hæmorrhage, cases of ...... 345

Cerebral hæmorrhage, lesions of motion from........ 350

Cerebral hæmorrhage, lesions of sensibility from ........ 352

Cerebral hæmorrhage, lesions of intelligence from ........ 357

Cerebral hæmorrhage, lesions of organic functions in ........ 359

Chest, on the physical examination of the ........ 70

Chloride of sodium, Dr. Graves on .. 489

Cholera, Dr. Spencer on............. 60

Chorea, as an epidemic in middle ages, 50

Clark, Dr. on phthisis.............. 1

Clendinning, Dr. statistical report.... 549

Clinical Course of M. Bouillaud, abstract of ... .................... 183

Clinical instruction, M. Louis essay on, 268

Clinique Medicale. Par M. Andral.. 89

Clinique Medicale, M. Andral's ..... 345

Cold and climate, Dr. Osborne on.... 508

Committee, report of, on the sounds of the heart...................... 491

Comparative Anatomy, Dr. Grant's Outlines of ........................ 376

Comparative anatomy of muscular system ........................... 380

Comparative anatomy of nervous system ........................... 381

Congenital defects, Dr. Hutton on .. 511

Constitution of man, Mr. Combe on the .......................... 361

Constitutional irritation, Mr. Travers on ............................. 297

Consumption, why so fatal? by Dr. Tyrrell ........................ 1

Convulsions des femmes, M. Velpeau on ............................ 61

Cornea, development of the ........ 251

Cornea, opacity of the ............ 252

Cornea, staphyloma of ............ 253

Coroner's inquest on an infant ...... 160

Crampton, Mr. on worms in the deer, 497

Creosote, composition of .......... 448

Creosote action of ................ 449

Creosote, medicinal employment of.. 450

Creosote, mode of exhibiting ...... 451

Curriculum of Apothecaries' Company, 289

Cutaneous diseases, eclectic article on, 414

Cutaneous diseases, M. Rayer on .... 414

Cutaneous diseases, Dr. Green on ... 414

Cutaneous diseases, article on, in Cyclopædia ........................ 414

Cutaneous diseases, advantages of baths in ........................ 415

Cutaneous diseases, advantages of venæsection in ................... 416

Cutaneous diseases, purgatives in. .. 417

Cutaneous diseases, alkalies and acids in............................ 418

Cutaneous diseases, mercury in...... 419

Cutaneous diseases, arsenic in ...... 420

Cutaneous diseases, iodine in ...... 421

Cutaneous diseases, classification of.. 422

D.

Defæcation, new views of.......... 508

Delirium tremens, Dr. Cross on .... 57

Dendy, Mr. his oration ............ 153

Depletion, inordinate, bad effects of. 475

Diabetes, clinical report on ........ 542

Diabetes, dissection of a case........ 548

Diagnosis, Mr. Stokes on .......... 501

Diffuse inflammation of the throat .. 328

Dislocation of os humeri ......... 525

Dissection-wound, fatal. .......... 445

Donovan, Dr. on urine in diabetes .. 543

Dropsy, methodical compression in .. 223

Dropsy, Dr. Carbutt's clinical report on .............................. 272

Dropsy, causes of ................. 272

Dropsy, passive and active......... 272

Dropsy, treatment of .............. 275

Dropsy after scarlatina ............ 429

Dropsy, Dr. Graves' case of ........ 446

Dropsy, renal, Mr. Anderson on .... 537

Ducts, biliary, occlusion of the...... 452

Dunbar, Dr. his galvanic experiments, 146

Dura mater, suppuration of ........ 560

E.

Economy, philanthropic, Mrs. Loudon, 576

Eczema, chronic, remarks on ...... 432

Edinburgh Infirmary, Mr. Syme's report ........................... 560

Egg's truss ....................... 513

Elephantiasis, Greek and Arabian.... *436

Encephaloid disease of the lungs .... 75

English surgery, French opinions on.. 266

Enteritis, case of.................. 166

Entozoa in human muscles ........ 496

Epidemics of the middle ages ...... 49

Erysipelas, report on .............. 196

Erysipelas, Mr. Travers on.......... 315

Erysipelas of the mucous membranes, 320

Erysipelas, and other cutaneous inflammations ................... 322

Erysipelas, what is it? ............ 323

Erysipelas, causes of .............. 324

Erysipelas, treatment of............ 326

Erysipelas, contagiousness of? ...... 327

Erysipelas of the throat............ 328

Erysipelas, remarks on ............ 426

Erythema nodosum, Mr. Blackley on.. 466

Erythema hæmorrhagica, Mr. Blackley on ........................ 469

Exanthemata, Rayer, &c. on the .... 423

Expectoration, as a symptom ...... 73

Experiments on the 2d sound of the heart ........................ 82

Extractor, obstetric, Dr. Breen on the, 454

F.

Fever, gastro-enteric, report on..... 197

Fever, chloride of sodium in ........ 489

Fever, desperate case of............ 529

Fever, yellow, Dr. Madden on ...... 575

Fish, prejudices against ............ 165

Fœtus, morbid anatomy of the...... 139

Fœtus, apoplexy of the ............ 140

Fœtus, apoplexy of brain in ........ 142

Fœtus, diseases of lungs and thymus of, 172

Fœtus, diseases of mouth and pharynx of .................... 173

Fœtus, follicular ulceration of stomach of .................... 173

Fœtus, diseases of spinal marrow of.. 173

Fœtus, case of monstrosity ........ 175

Fœtus, Mr. Johnson on venereal affections of the.................. 277

Fœtus, cases of venereal affection of.. 278

Follicular enteritis, M. Andral on ... 216

Fractures, Mr. Hinde's plates of .... 487

Fractures, absorption of callus in.... 523

G.

Galvanic experiments, Dr. Dunbar's.. 146

Gastro-enteritis, M. Andral on ...... 216

Gastrodynia, Dr. Osborne on ...... 562

Gonorrhœa, on the management of .. 177

Gonorrhœa, its supposed analogy to erysipelas .................... 178

Gonorrhœa, its nature ............ 179

Gonorrhœa, its actual phenomena.... 180

Gonorrhœa, treatment of .......... 181

Gonorrhœa, varieties of the discharge in .......................... 181

Gonorrhœa, painful erections in .... 182

Gonorrhœa, employment of mercury in.......................... 183

Gonorrhœa, copaiba and cubebs for.. 184

Gonorrhœa, Mr. Johnson's case of secondary symptoms after.......... 191

Gonorrhœal ophthalmia, treatment of, 228

Grant, Dr. his Outlines of Comparative Anatomy ................. 376

Graves, Dr. his case of dropsy ...... 446

Graves, Dr. on chloride of sodium .. 489

Graves', Dr. concluding address to the British Association.............. 512

Graves, Dr. on fever .............. 529

Greene, Dr. on the stethoscope in the diagnosis of thoracic aneurism.... 477

H.

Harrison, Dr. on bones in the ox's heart ...................... 495

Harveian Oration ................ 577

Headache, Dr. Weatherhead on...... 370

Headache, varieties of ............ 371

Headache, hysterical .............. 373

Headache, treatment of........... 373

Headache, arthritic................ 375

Heart, *immediate* cause of the sounds of the ...................... 80

Heart, experiments on the 2d sound of, 82

Heart, unique case of disease of the.. 152

Heart, diseased; value of physical signs shewn.................... 168

Heart, sounds of, report of committee, 498

Hernia, strangulated congenital...... 521

Hernia, Dr. Macfarlane on.......... 551

Hetling, Mr. on osteo-sarcoma...... 531

Hinde, Mr. his plates of fractures.... 487

History of Medicine, Dr. Bostock's .. 53

Homœopathy; scene in the French Academy ...................... 239

Hope, Dr. his appendix ............ 69

Hospitals (British), remarks on .... 569

Houston, Mr. on the circulation of the whale ........................ 490

Houston, Dr. on hydatids in the deer, 496

Humeri, os, dislocation of.......... 525

Hydatids, Dr. Houston on.......... 496

Hydrocephalus, M. Cruveilhier on .. 143

Hydrops pericardii, Dr. Lawson...... 248

Hyperæmia, cerebral, M. Andral's account of ...................... 96

I.

Iliac fossa, chronic abscess in the.... 258

Impetigo, remarks on.............. 434

Inflammation, subcutaneous ........ 519

Infusoria ........................ 403

Injuries of the abdomen............ 526

Insanity, Dr. Prichard on .......... 106

Insanity, in males and females ...... 107

Insanity, at different ages .......... 108

Insanity, causes of ................ 108

Insanity, morbid anatomy.......... 110

Insanity, state of the lungs in ...... 115

Insanity, treatment of.............. 116

Insanity, importance of seclusion in.. 119

Instinct, Mr. Kirby on ............ 401

Intermittents, masked, M. Andral on, 103

Intus-susception, treated by inflation, 164

Iritis, Mr. Watson on.............. 567

Irritation, constitutional, Mr. Travers on.......................... 297

Irritation, direct and reflected ...... 298

Irritation from pre-existing organic disease........................ 299

Irritation producing disease in distant parts.......................... 301

Irritation, direct and reflected, connexion of...................... 303

Irritation, shewn in local changes.... 307

Irritation in cachectic suppuration .. 310

Irritation in cachectic ulceration .... 310

Irritation in mercurial cachexia ..... 312

Irritation producing constitutional inflammations.................... 314

Irritation occasioning erysipelas ..... 315

J.

James, Mr. on good and bad stumps.. 45

Jaws, osteo-sarcoma of both ........ 532

Johnson, Dr. his case of diseased heart 168

Johnson, Mr. his case of secondary symptoms after gonorrhœa ... ... 191

Johnson, Mr. on venereal affections of the fœtus...................... 277

Johnson, Mr. on syphilitic sores .... 564

K.

Kennedy, Dr. on purulent ophthalmia, 503
Kirby, Dr. his Bridgewater Treatise .. 400
Kirkbride, Dr. on hernia .......... 522
Kreosote, Dr. Elliotson on.......... 151
Kreosote, Mr. Taylor on the use of .. 541

L.

Lacrymal caruncle, inflammation of the 254

Laryngitis, Dr. M'Adam's case of.... 472

Lecturers, modern glut of .......... 154

Legs, why prone to ulcers .......... 390

Lepra and psoriasis, Dr. Pennock on.. 457

Lepra and psoriasis, remarks on ..... 463

Lepra and psoriasis, treatment of .... 464

Lichen, M. Rayer on the treatment of, 437

Louis, M. on phthisis.............. 1

Louis, M. on blood-letting in pneumonia .......................... 200

Louis, M. his essay on clinical instruction .......................... 268

Lungs, perforation of, in phthisis.... 20

M.

Mac Adam, Dr. his case of laryngitis, 472

M'Donnell, Dr. on the heart and pulse 493

M'Farlane, Dr. on hernia .........., 551

M'Leod, Dr. clinical report from .... 558

Madden, Dr. on the West Indies .... 574

Mamma, male, affections of the...... 255

Man, Mr. Combe on .............. 361

Mary-le-bone Infirmary, report from, 550

Maton, the late Dr. character of .... 577

Medical Society of New York, Transactions of......................... 57

Medical tuition, circular of the officers of St. Bartholomew's Hospital .... 162

Medical reform .................... 286

Medicine, Dr. Bostock's history of .. 53

Medullary disease of eye-ball........ 560

Mercury in continued fever, Dr. M'Leod on ............................ 559

Methodical compression in dropsy.... 223

Middlemore, Mr. his ophthalmic report 251

Mirage, Lieut. Burns' description of the 163

Molluscæ, Mr. Kirby on the........ 408

Monomania, case of .............. 150

Morbid anatomy of the fœtus ....... 139

Mortification from cessation of circulation ......................... 123

Mortification from inflammation .... 123

Mortification from mechanical obstruction to the circulation ........... 131

Mortification from debility.......... 134

Mortification from chemical and other agents ......................... 135

Mortification from poisons ......... 137

Muscular system, comparative anatomy of........................... 380

N.

Nervous system, comparative anatomy of the ....................... 381

O.

O'Beirne, Dr. on defæcation......... 508

Obstetric extractor, Dr. Breen on the, 454

Occlusion of the vagina, case of ..... 474

Œsophagitis, case of .............. 166

Omentum, disease of the .......... 261

Ophthalmia, gonorrhœal, treatment of 228

Ophthalmia, purulent treatment of .. 232

Ophthalmia, purulent, Dr. Kennedy on 503

Osborne, Dr. on diseases of the stomach ......................... 562

Osteo-sarcoma, dreadful case of ..... 531

Outlines of comparative anatomy, by Dr. Grant......................... 376

Ovarian tumors, Dr. Macfarlane on .. 264

P.

Paraplegia, curious case of......... 160

Pathological anatomy, Dr. Carswell on 122

Pathological anatomy, M. Cruveilhier on ............................ 122

Pennock, Dr. on lepra and psoriasis.. 457

Pericarditis, obscure case of ........ 156

Pericarditis, rheumatic ............. 250

Pericardium, Dr. Law on dropsy of the 248

Pertussis, cured by revulsives ...... 215

Philip, Dr., Gulstonian Lectures .... 515

Phthisis, M. Louis on.. .... ...... 1

Phthisis, Dr. Clark on ........ .... 1

Phthisis, why so fatal? by J. Tyrrell, Gent. ......................... 1

Phthisis, morbid anatomy of ........ 5

Phthisis, state of pleuræ in ........ 8

Phthisis, state of trachea in ........ 9

Phthisis, state of larynx in.......... 10

Phthisis, state of epiglottis in ....... 11

Phthisis, state of heart in .......... 12

Phthisis, state of digestive system in.. 12

Phthisis, large intestine in.......... 15

Phthisis, state of liver in ......... 17

Phthisis, state of spleen and kidneys in 18

Phthisis, perforation of the lungs in.. 20

Phthisis, the more marked varieties of, 25

Phthisis, latent, cases of .......... 28

Phthisis, acute form of ............ 32

Phthisis, chronic form of .......... 40

Phthisis, bronchial, description of.... 42

Physical signs of diseases of the chest, Dr. Williams on the chest ....... 69

Physical signs of disease of heart, case shewing the value of ........... 168

Placenta, diseases of the............ 176

Placenta, hypertrophy of .......... 176

Placenta, atrophy of .............. 176

Placenta, inflammation of .......... 176

Placenta, ossification of the ........ 177

Placenta, apoplexy of the ......... 177

Pleurisy, chronic, with pneumo-thorax 206

Pneumo-thorax and phthisis, case of.. 187

Pneumonia, effects of blood-letting in, 200

Pneumonia, typhoid, Dr. Hudson on, 485

Polypes ........................ 405

Porrigo, remarks on ............... 435

Prichard, Dr. on insanity .......... 106

Provincial Medical Association, Transactions of. .................. 45

Puerperal fever, Dr. Eights on ...... 59

Puerperal convulsions, M. Velpeau on, 61

Puerperal convulsions, partial ...... 62

Puerperal convulsions, hysteric...... 65

Puerperal convulsions, tetanic ...... 65

Puerperal convulsions, epileptic ..... 65

Puerperal convulsions, apoplectic.... 66

Puerperal convulsions, treatment of.. 67

Pulse, Dr. M'Donnell on the........ 493

Purulent ophthalmia, treatment of .. 232

Q.

Quain, Dr. anatomical plates ...... 576

Quarterly Medical Review, obituary of ........................... 579

R.

Radiaries, Mr. Kirby on the ........ 406

Renal dropsy, Mr. Anderson on ..... 537

Respiratory apparatus, diseases of the, 183

Rheumatism, acute, tartrate of antimony in ...................... 235

Rupia, remarks on ....... ........ 430

S.

Sac, sloughing of, in hernia ........ 522

St. George's Hospital, new laws of .. 281

Salmon's self-adjusting truss........ 514

Sangaree, Dr. Madden on .......... 575

Scaly diseases, classification of the .. 440

Scaly diseases, treatment of ........*434

Secondary symptoms after gonorrhœa, Mr. Johnson's case of............ 191

Sleep, Mr. Carmichael on the cause of, 470

Sloughing of the anus ............ 527

Spender, Mr. on ulcers of the legs.... 388

Sphygmometer, its absurdity........ 159

Spitting of blood, its occasional slight cause ........................ 150

Statistical report from the Birmingham Infirmary...................... 241

Statistical report from St. George's Hospital ...................... 558

Statistics of Marylebone............ 549

Stethoscope in the diagnosis of thoracic aneurism ................. 477

Stokes, Dr. on points of diagnosis.... 501

Stomach and liver, displacement of .. 187

Stroud, Dr. his cases of carditis ..... 441

Stumps, Mr. James on the management of ...................... 45

Subcutaneous inflammation ........ 519

Sugar and starch in diabetic urine.... 544

Syme, Mr. clinical report from ...... 560

Syphilitic sores, Mr. Johnson on ..... 564

T.

Tarantism in Italy .. ............. 51

Taylor, Mr. on kreosote............ 541

Thoracic diseases, Dr. Stokes on .... 502

Transactions of the Provincial Association ....................... 45

Transactions of Medical Society of New York ................... 57

Traumatic tetanus cured ........... 484

Travers, Mr. on constitutional irritation ......................... 297

Tropical night, miseries of.......... 575

Trusses, on the varieties of, Mr. Acret, 512

Tumor of the thigh, obscure case of.. 282

Tumors of the abdomen, report on .. 254

Typhoid pneumonia, Dr. Hudson on.. 485

U.

Ulcers of the legs, Mr. Spender on .. 388

Ulcers of the legs, treatment of...... 394

Urethra, syphilitic sores in.......... 564

Urine, constituents of, in diabetes.... 534

Urticaria, M. Rayer on ............ 428

V.

Vagina, case of occlusion of the...... 474

Varicose ulcers, Mr. Spender on .... 392

Velpeau on the puerperal convulsions, 61

Venereal affections of the fœtus, Mr. Johnson on the ................. 277

Vertebræ, elemental constituents and development of ............... 377

Vision, temporary loss of .......... 528

Vomica cured by bleeding? ........ 213

W.

Waddell, Dr. his case of paraplegia .. 160

Wallace, Mr. on gonorrhœa ........ 177

Watson, Mr. report from eye-infirmary 566

Weatherhead, Dr. on headaches ..... 370

Whale, the circulation in the ....... 490

Whatton, Mr. on partial amputation of foot ...................... 499

Williams, Dr. on the physical signs of diseases of the chest ........... 69

### ST. GEORGE'S HOSPITAL.

The WINTER COURSES of LECTURES will commence on *October* 1*st*.

THEORY and PRACTICE of PHYSIC—by Dr. MACLEOD and Dr. SEYMOUR.

THEORY and PRACTICE of SURGERY—by Mr. CÆSAR HAWKINS and Mr. G. BABINGTON.

MATERIA MEDICA—by Dr. SEYMOUR and Dr. MACLEOD.

MIDWIFERY and DISEASES of WOMEN & CHILDREN—by Dr. R. LEE, F.R.S.

MEDICAL JURISPRUDENCE—by Dr. HOPE, F.R.S.

BOTANY—by Dr. DICKSON.

CLINICAL LECTURES on MEDICINE—by Dr. SEYMOUR & Dr. MACLEOD.

CLINICAL LECTURES on SURGERY—by SIR BENJAMIN BRODIE, Bart., Mr. C. HAWKINS, and Mr. G. BABINGTON.

*A Library, Museum, Herbarium, and Collection of Materia Medica, are provided for the use of the Students.*

---

### THEATRE OF ANATOMY,

*Kinnerton Street, Wilton Place, adjoining* St. George's Hospital.

A COURSE OF LECTURES ON ANATOMY, PHYSIOLOGY, AND SURGICAL ANATOMY, will be delivered at this School, by

Mr. TATUM,

*Formerly Teacher of Anatomy at the School of Great Windmill Street, and House Surgeon to St. George's Hospital; and*

Mr. HENRY JAMES JOHNSON,

*Formerly House Surgeon to St. George's and the Lock Hospitals.*

A Lecture will be given daily at half-past Two o'clock, during the Season. The Lectures will commence on the 1st of *October*, and terminate about the middle of the ensuing *May*.

*The Operations of Surgery will be shewn during the latter part of the Course.*

A COURSE OF DEMONSTRATIONS OF PRACTICAL ANATOMY will be delivered by

Mr. HENRY JAMES JOHNSON, and Mr. HENRY CHARLES JOHNSON, *Curator of the Museum of St. George's Hospital, and formerly House Surgoon.*

A Demonstration will be given daily, during the Course, at half-past Ten o'clock in the morning.—The Demonstrations will commence on the 10th of *October*, and terminate early in the ensuing *May*.

*One Morning in the Week will be exclusively devoted to examination of the Class.*

### TERMS:

| | £. | s. | d. |
|---|---|---|---|
| LECTURES. | | | |
| One Course* | 6 | 6 | 0 |
| Perpetual | 8 | 8 | 0 |
| DEMONSTRATIONS. | | | |
| One Course | 6 | 6 | 0 |
| Perpetual | 8 | 8 | 0 |
| Perpetual to Lectures and Demonstrations | 16 | 16 | 0 |

* One Course both of Lectures and Demonstrations, now extends through the whole Anatomical Session; viz. from *October* to *May*.

Gentlemen desirous of further information, may obtain it by applying at the School—or at the Residences of the Teachers, Mr. Tatum, 7, *Berkeley-street, Berkeley-square;* Mr. Henry Jas. Johnson, 8, *Suffolk-place, Pall Mall East;* Mr. Henry Charles Johnson, 3, *New Burlington-street.*

